# Supplementary material for: Estimating body volumes and surface areas of animals from cross-sections
Source: PeerJ. 2024 May 28;12:e17479. doi: 10.7717/peerj.17479 (PMC11141563; doi:10.7717/peerj.17479)
Supplement: Supplemental Information 2 [file peerj-12-17479-s002.pdf]

Estimating body volumes and surface areas of animals from cross-sections

Supplementary Material: Reconstruction of *Tyrannosaurus rex* AMNH 5027

Ruizhe Jackevan Zhao[1]
[1] Department of Mathematics, Northwest University, Xi'an, Shaanxi, China

Corresponding Author:
Ruizhe Jackevan Zhao[1]

Email address: jackevanchaos@outlook.com

Body Length

The skull of AMNH 5027 was reproduced from the illustration by Carr (2020). Length from the tip of the snout to basioccipital condyle is 1.21 m (Osborn, 1912).

The precaudal vertebral column was reproduced from (Osborn, 1917). The tail region of AMNH 5027 is incompletely preserved, and Osborn (1917) restored 53 caudals for the mount. This number is unrealistic for *T. rex* (Brochu, 2003), and the tail of the mount is too long. Length of the tail region was therefore calculated using the ratio of precaudal length to tail length in FHSM PR 2081 (pers. orbs. from the 3D scanned mount). This leads to an 11.2 m estimate for body length along the vertebral column of AMNH 5027.

Skull Length	Precaudal Length	Tail Length	Total Length
1.210 m	5.316 m	5.912 m	11.228 m

Body cross-sections

Cross-section of the skull was summarized from the posterior view illustration (Osborn, 1912: Fig. 4). Three cross-sections were reconstructed for the ribcage, which are in the levels of D1, D4 and D11 respectively. Slant angles of dorsal ribs from side view and dorsal view were

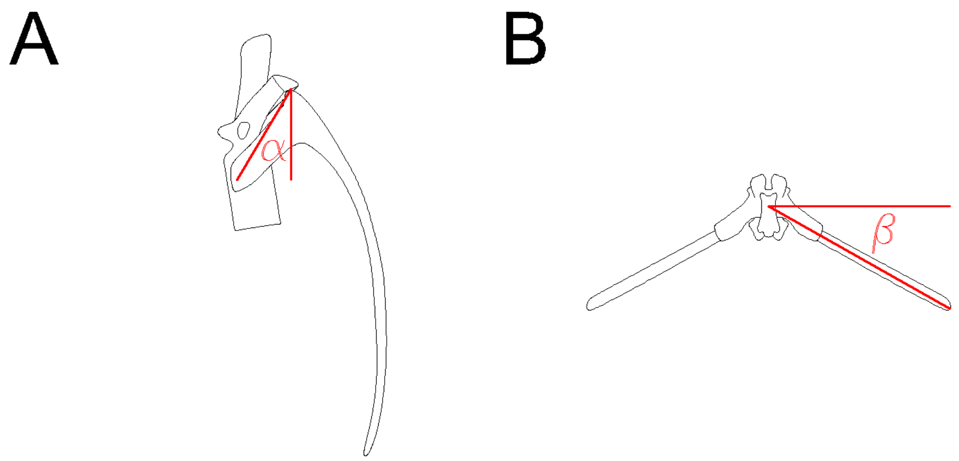

Figure 1. Illustration showing the two slant angles affecting the rib orientation in *T. rex*. (A) Slant angle (α) from left side view. (B) Slant angle (β) from dorsal view.

summarized from the 3D model scanned from the mount. Hirasawa (2009) studied ligamental scars and proposed how the ribs should be articulated with corresponding transverse processes in tyrannosaurids. It is notable that some ribs in the mount were not assembled in the way proposed by Hirasawa (2009), hence they were truncated from the 3D model, then redrawn and rearranged in AutoCAD.

D4 is used here as an example to show how the ribs were arranged. There are two slant angles affecting the orientation of each rib in *T. rex:* one from side view and the other from dorsal view (Fig. 1). The rib was first placed in a 2D plane attached to the corresponding dorsal vertebra (the black one on the left of Fig. 2). Then its maximum width and height were measured and multiplied by cosine values of corresponding slant angles (a gray schematic rib is shown in Fig. 2). Finally, a smooth curve representing body outline is drawn along the corrected rib.

Three cross-sections were reconstructed for the tail. The first one truncates the posterior end of the ischia and is in the level of the first caudal. A short distance behind, a second cross-section was created, representing the body outline that encases the tail. The third cross-section was set between caudal 17 and 18 since transverse processes behind this level are small in size or entirely absent from the vertebrae (Brochu, 2003). Persons and Currie (2011) argued that the tail muscles have a significant impact on the location of the center of mass, hence the tail cross-sections of AMNH 5027 were reconstructed following their criteria.

One cross-section was created for the hindlimbs, reproduced from the dorsal view skeleton of *T. rex* in (Paul, 1988).

Volume Calculation

The main body was partitioned into 8 slabs by the 7 cross-sections, and each hindlimb was

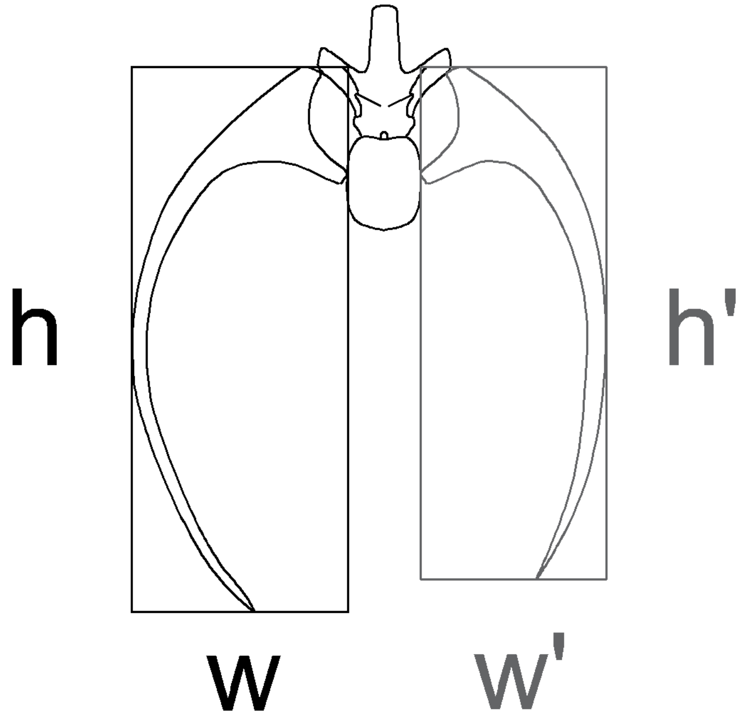

Figure 2. Illustration showing how the rib cage can be restored in 2D environment. The black one on the left is the rib placed on a horizontal plane, while the gray one on the right is the schematic rib in the three-dimensional ribcage. Here $h' = h \times \cos\alpha$, $w' = w \times \cos\beta$, where α and β are slant angles shown in Figure 1.

regarded as one slab with constant cross-sections. Each slab was further divided into 1000 subslabs, and the CSM was applied to calculate their volumes. Soft outlines of the forelimbs

and toes are unknown; hence they were approximated using cylinders. This would not cause significant influence in the total volume due to their small sizes.

References

Brochu CA. 2003. Osteology of Tyrannosaurus rex: Insights from a nearly complete Skeleton and High-Resolution Computed Tomographic Analysis of the Skull. *Journal of Vertebrate Paleontology* 22:1–138. DOI: 10.1080/02724634.2003.10010947.

Carr TD. 2020. A high-resolution growth series of Tyrannosaurus rex obtained from multiple lines of evidence. *PeerJ* 2020. DOI: 10.7717/peerj.9192.

Hirasawa T. 2009. The ligamental scar in the costovertebral articulation of the tyrannosaurid dinosaurs. *Acta Palaeontologica Polonica* 54:49–59. DOI: 10.4202/app.2009.0106.

Osborn HF. 1912. Crania of Tyrannosaurus and Allosaurus. *Memoirs of the American Museum of Natural History*.

Osborn HF. 1917. Skeletal adaptations of Ornitholestes, Struthiomimus, Tyrannosaurus.No Title. *Bulletin of the AMNH*.

Paul GS. 1988. *Predatory dinosaurs of the world: a complete illustrated guide*.

Persons WS IV & Currie Philp J. 2011. The tail of Tyrannosaurus: reassessing the size and locomotive importance of the M. caudofemoralis in non-avian theropods. *Anatomical Record: Advances in Integrative Anatomy and Evolutionary Biology* 294(1):119–131.
